# Postprandial changes of lipoprotein profile: effect of abdominal obesity

**DOI:** 10.1186/1476-511X-12-179

**Published:** 2013-12-08

**Authors:** Peter Sabaka, Peter Kruzliak, Ludovit Gaspar, Martin Caprnda, Matej Bendzala, David Balaz, Stanislav Oravec, Andrej Dukat

**Affiliations:** 1 Department of Internal Medicine, University Hospital in Bratislava and Faculty of Medicine in Bratislava, Comenius University in Bratislava, Bratislava, Slovak Republic; 2 Department of Cardiovascular Diseases, International Clinical Research Center, St. Anne’s University Hospital and Masaryk University, Pekarska 53, 656 91 Brno, Czech Republic

**Keywords:** Postprandial, Lipoprotein subclasses, Abdominal obesity, Electrophoresis

## Abstract

**Background:**

Majority of studies that focused on the influence of abdominal obesity on lipoprotein profile, were conducted in the fasting conditions. The effects of visceral fat accumulation on postprandial lipoprotein concentrations have not yet been studied in details. We therefore focused on the postprandial lipoprotein profile in otherwise healthy men and women with abdominal obesity and their comparison with the control group of volunteers with normal waist circumference. The concentration of lipoprotein classes and subclasses was measured before and 4 hours after a standard meal by linear polyacrylamide gel electrophoresis.

**Results:**

A statistically significant postprandial rise in triacylglycerol concentration occurred in all subjects. VLDL increased 4 hours after meal in all subjects except the women with normal waist circumference. The concentration of large IDL particles increased in both non-obese men and women. In women with abdominal obesity, however, it decreased, while in obese men there was no statistically significant change. The concentration of small and medium-sized IDL particles decreased in all volunteers. Analyzing subclasses changes of large, medium-sized and small LDL particles we saw no significant shift in their concentrations except the subclass of large LDL particles, which decreased in men. Concentrations of medium and small HDL particles decreased postprandially in all volunteers with normal waist circumference. However, they remained unchanged in subjects with abdominal obesity.

**Conclusions:**

We observed significant postprandial changes of the lipoprotein profile, but the nature and extent of these changes depended on gender and presence of abdominal obesity.

## Background

Most of our knowledge about plasma lipoproteins and their relation to the risk of developing cardiovascular diseases as well as their relations to other cardiovascular risk factors is based on the finding of studies conducted in fasting conditions [1,2]. The metabolism of lipoproteins, however, is in a fasting state for only a small part of the day. In developed countries an average individual consumes daily 3 or more meals, each containing 20 to 70 grams of fat. In such food intake model, the individual is mostly in the postprandial state. The fasting metabolic state comes only after longer night-time fasting period [3]. The study of postprandial changes of the concentrations of lipoprotein profile is of great importance in clarifying the physiological and pathophysiological mechanisms in the postprandial period. It also may provide us with a picture of the lipoprotein profile in the metabolic state which is present the most of the day [3,4]. As we know so far, the food intake has the greatest effect on acute rise of triacylglycerol rich lipoproteins. It is also known that high postprandial triacylglycerolemia is strong predictor of myocardial infarction and ischemic heart disease [5]. Studies yet published recorded postprandial rise of triacylglycerol, chylomicron, VLDL and IDL concentrations. These studies described also some changes in concentrations of LDL and HDL, but their magnitude were much less extensive [6,7]. The LDL and HDL particles, however, do not present a homogeneous lipoprotein groups, but they are divided by size and density into lipoprotein subclasses of small, medium and large particles. These subclasses differ in biological characteristics and also in their atherogenic potential [8,9]. Only a small number of studies have been devoted to postprandial changes in the LDL and HDL lipoprotein subclasses. These studies also differ in methodology used both to determine lipoproteins and the design of the study itself [6,10-13]. Our knowledge of the postprandial changes of these lipoprotein subclasses is therefore fairly incoherent. The effect of the lipid rich meal on the concentrations of IDL particle subclasses has not been described so far. In the light of the current knowledge it appears that the abdominal obesity and the associated insulin resistance significantly affect the postprandial lipoprotein metabolism [14]. Abdominal obesity is well accepted cardiovascular risk factor [15]. Nevertheless, the differences in postprandial changes of lipoprotein concentrations between individuals with abdominal obesity and individuals with normal waist circumference have not been compared yet. These differences may lead to better understanding of formation of atherogenic lipoprotein profile in these subjects.

We studied the acute impact of high fat meal on the concentrations of serum lipoprotein classes and subclasses in healthy men and women as well as men and women with abdominal obesity. Our goal was to identify changes in concentrations of these lipoproteins in the postprandial period including IDL subclasses and to compare the differences in postprandial changes of lipoprotein profile between individuals with abdominal obesity and the control group.

## Results

### Population characteristics

The study included 48 volunteers, of whom 24 were women and 24 were men. The cohort was split into two groups according to the presence of abdominal obesity (12 men and 12 women with abdominal obesity and 12 men and 12 women without abdominal obesity). Waist circumference higher than 94 cm in men and 80 cm in women was used for diagnosis of abdominal obesity, according to IDF criteria. Age, BMI and waist circumference for each cohort of tested individuals are shown in Tables 1 and 2.

**Table 1:** Cohort description - men

**Cohort**	**Control**	**Abdominal obesity**	**P (waist)**
Age	24.44 ± 2.351	31.00 ± 10.360	0.142
BMI (kgm^-2^)	21.22 ± 0.833	28.11 ± 1.364	< 0.0001
Waist (cm)	78.22 ± 4.868	104.40 ± 7.56	< 0.0001
TAG fasting (mg/dl)	101.06 ± 63.956	136.46 ± 31.496	< 0.05
TAG postprandial (mg/dl)	162.12 ± 51.708	294.78 ± 111.239	< 0.01
VLDL fasting (mg/dl)	29.25 ± 13.57	34.22 ± 8.758	< 0.05
VLDL postprandial (ml/dl)	32.50 ± 11.43	40.89 ± 9.453	< 0.05

**Table 2:** Cohort description - women

**Cohort**	**Control**	**Abdominal obesity**	**P (waist)**
Age	24.12 ± 2.108	27.78 ± 3.701	0.276
BMI (kgm^-2^)	22.00 ± 2.028	28.33 ± 4.822	< 0.0001
Waist (cm)	68.11 ± 4.658	97.78 ± 5.783	< 0.0001
TAG fasting (mg/dl)	76.50 ± 64.690	79.92 ± 45.929	< 0.05
TAG postprandial (mg/dl)	121.06 ± 88.487	133.63 ± 72.212	0.667
VLDL fasting (mg/dl)	23.33 ± 8.646	23.44 ± 8.233	0.537
VLDL postprandial (mg/dl)	24.78 ± 9.324	27.56 ± 7.552	< 0.05

### Triacylglycerols

**Figure 1:**
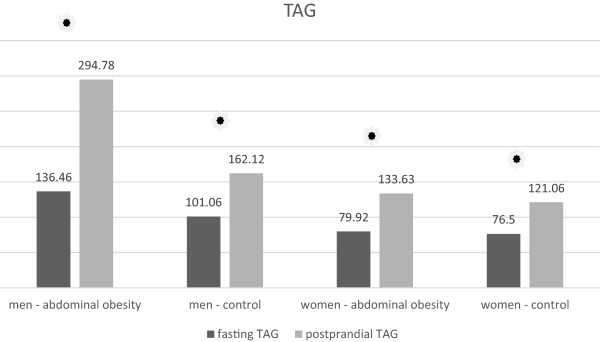
Fasting and postprandial TAG concentrations.

The men with abdominal obesity had significantly higher basal triacylglycerol (TAG) concentrations than men with normal waist circumference as well as the both women cohorts (Table 3, Figure 1). We observed the same pattern in postprandial concentrations of TAG (Table 4, Figure 1). Postprandial increase in TAG concentration occurred in all groups of the volunteers (Tables 5 and 6, Figure 1). The most significant rise of the average concentration of TAG was observed in the group of men with abdominal obesity (Table 5). In this group, the average value of TAG more than doubled and the rise was significantly higher than in the group of men without abdominal obesity (Table 6). The magnitude of rise in the TAG concentration in the women with abdominal obesity did not significantly differ from the rise of TAG in women with normal waist circumference (Table 6).

**Table 3:** Fasting TAG in mg/dl

**Cohort**	**Men**	**Women**	**P (gender)**
Control	101.06 ± 63.956	76.50 ± 64.690	0.144
Abdominal obesity	136.46 ± 31.496	136.46 ± 31.496	< 0.01
P (waist)	< 0.05	< 0.05	

**Table 4:** Postprandial TAG in mg/dl

**Cohort**	**Men**	**Women**	**P (gender)**
Control	162.12 ± 51.708	121.06 ± 88.487	< 0.01
Abdominal obesity	294.78 ± 111.239	133.63 ± 72.212	< 0.005
P (waist)	< 0.005	0.667	

**Table 5:** Postprandial changes of TAG and VLDL in men

**Lipoprotein**	**Cohort**	**Fasting**	**Postprandial**	**P (time)**	**P (time × waist)**
TAG (mg/dl)	Control	101.06 ± 63.956	162.12 ± 51.708	< 0.01	< 0.005
	Abdominal obesity	136.46 ± 31.496	294.78 ± 111.239	< 0.01	
VLDL (mg/dl)	Control	29.25 ± 13.570	32.50 ± 11.43	< 0.05	0.178
	Abdominal obesity	34.22 ± 8.758	40.89 ± 9.453	< 0.01	

**Table 6:** Postprandial changes of TAG and VLDL in women

**Lipoprotein**	**Cohort**	**Fasting**	**Postprandial**	**P (time)**	**P (time × waist)**
TAG (mgl/dl)	Control	76.50 ± 64.690	121.06 ± 88.487	< 0.05	0.313
	Abdominal obesity	136.46 ± 31.496	133.63 ± 72.212	< 0.005	
VLDL (mg/dl)	Control	23.33 ± 8.646	24.78 ± 9.324	0.553	< 0.05
	Abdominal obesity	23.44 ± 8.233	27.56 ± 7.552	< 0.05	

### VLDL

**Figure 2:**
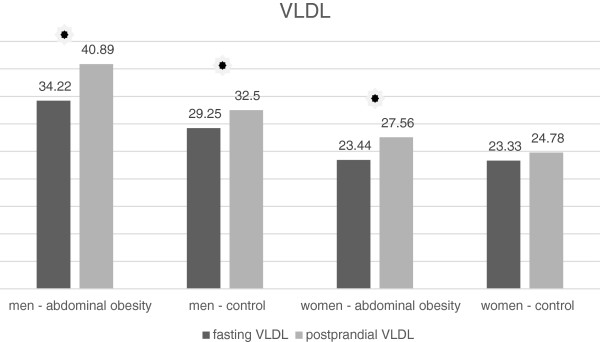
Fasting and postprandial VLDL concentrations.

Mean fasting VLDL concentration was significantly higher in the group of men with abdominal obesity than in control group (Table 1, Figure 2). However there was no significant difference between the obese and non-obese women (Table 2, Figure 2). Postprandial VLDL concentrations were significantly higher in men and also in women with abdominal obesity (Tables 1 and 2). In both groups of men and in women with abdominal obesity, the postprandial increase of VLDL was recorded (Tables 5 and 6, Figure 2). Postprandial VLDL concentration in men with abdominal obesity was significantly higher than in the non-obese men, however the rate of postprandial increase between these cohorts was not significantly different (Table 5). In women with a normal waist circumference a statistically significant rise of VLDL has not been recorded (Table 6).

### IDL and their subclasses

**Figure 3:**
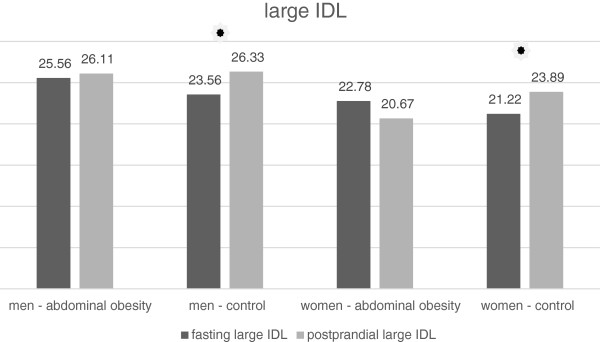
Fasting and postprandial large IDL concentrations.

**Figure 4:**
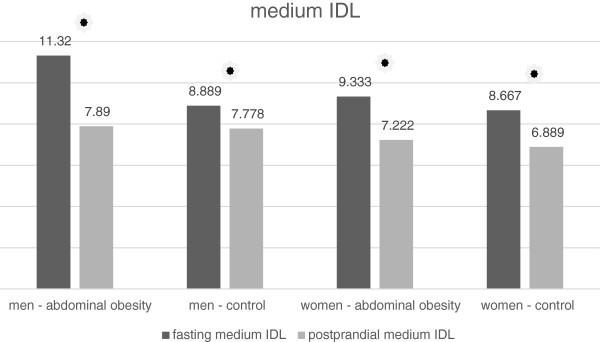
Fasting and postprandial medium IDL concentrations.

**Figure 5:**
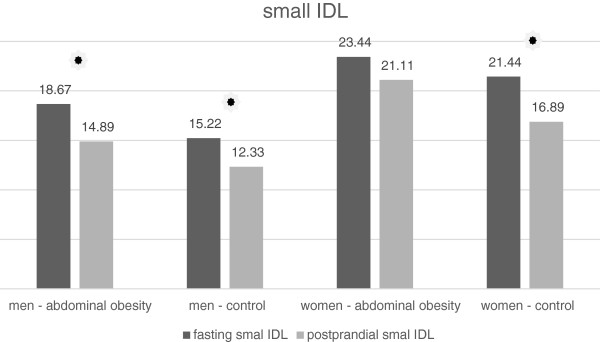
Fasting and postprandial small IDL concentrations.

In men and women with normal waist circumference, there was a small but statistically significant increase in the concentration the lipoprotein subclass, referred to as MID C by the Quantimetrics Lipoprint. This subclass represents large IDL particles (Tables 7 and 8, Figure 3). By contrast, in the group of obese women, there was a statistically significant decrease in the concentration of these particles (Table 8, Figure 3). In the obese men there was any significant change in the MID C concentration (Table 7, Figure 3). In the MID B subclass which represents medium-sized IDL particles we noticed a small but statistically significant decrease in the average concentration after the meal in all groups of volunteers. Decrease in the concentration was significantly higher in men with abdominal obesity than in non-obese men (Table 7, Figure 4). In women, there was no statistically significant difference between obese and non-obese subjects (Table 8, Figure 4). Similarly, a decrease in the average concentration of MID A has been established in all groups except the group of women with abdominal obesity (Tables 7 and 8, Figure 5). In this group the decrease was at borderline significance (p = 0.057). This subclass consists of small IDL particles.

**Table 7:** Postprandial changes of IDL subclasses in men

**Lipoprotein**	**Cohort**	**Fasting**	**Postprandial**	**P (time)**	**P (time × waist)**
MID C (mg/dl)	Control	23.56 ± 4.447	26.33 ± 4.387	< 0.01	< 0.01
	Abdominal obesity	25.56 ± 4.773	26.11 ± 4.807	0.608	
MID B (mg/dl)	Control	8.89 ± 2.205	7.78 ± 2.728	< 0.05	< 0.05
	Abdominal obesity	11.32 ± 3.279	7.89 ± 2.147	< 0.01	
MID A (mg/dl)	Control	15.22 ± 2.819	12.33 ± 1.225	< 0.05	0.140
	Abdominal obesity	18.67 ± 4.77	14.89 ± 2.892	< 0.01	

**Table 8:** Postprandial changes of IDL subclasses in women

**Lipoprotein**	**Cohort**	**Fasting**	**Postprandial**	**P (time)**	**P (time × waist)**
MID C (mg/dl)	Control	21.22 ± 6.300	23.89 ± 4.885	< 0.05	< 0.01
	Abdominal obesity	22.78 ± 4.177	20.67 ± 3.428	< 0.05	
MID B (mg/dl)	Control	8.667 ± 3.279	6.889 ± 3.333	< 0.05	0.426
	Abdominal obesity	9.333 ± 3.775	7.222 ± 3.383	< 0.01	
MID A (mg/dl)	Control	21.44 ± 12.98	16.89 ± 9.816	< 0.05	0.099
	Abdominal obesity	23.44 ± 8.604	21.11 ± 7.474	0.057	

### LDL and their subclasses

**Figure 6:**
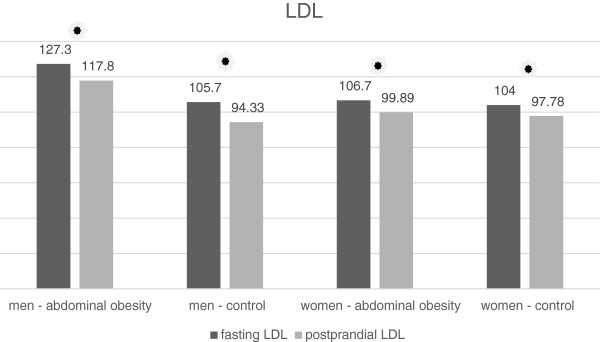
Fasting and postprandial total LDL concentrations.

**Figure 7:**
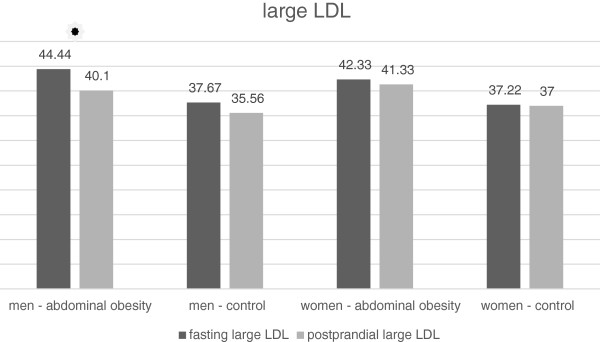
Fasting and postprandial large LDL concentrations.

**Figure 8:**
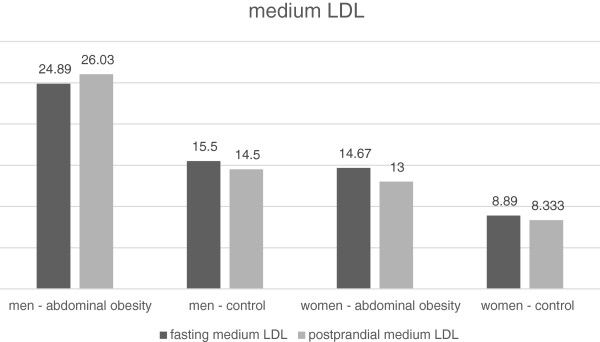
Fasting and postprandial medium LDL concentrations.

**Figure 9:**
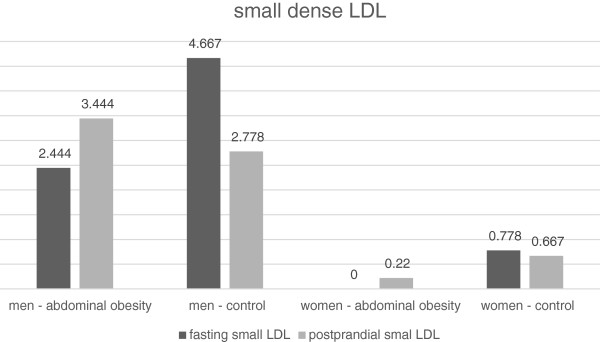
Fasting and postprandial small dense LDL concentrations.

The LDL concentration after the meal decreased significantly in all groups of the volunteers (Tables 9 and 10, Figure 6). There was a decrease in the concentration of the large LDL particle subclass (LDL 1) both in obese and non-obese men (Table 9, Figure 7). On the contrary, we have not recorded any significant change in women (Table 10, Figure 7). There was no statistically significant change in the concentrations of medium-sized (LDL 2) and small (LDL 3 to 7) LDL particles in any of the study groups (Tables 9 and 10, Figures 8 and 9). However, the results in the case of small LDL particles are not relevant, since only 7 men and 3 women from the 40 volunteers had detectable concentrations of small LDL particles. It is the reduction of the MID A and MID B lipoprotein fractions, which is mainly responsible for the decrease in the LDL concentration in the study population. By the Quantimetrics Lipoprint LDL system the MID A and MID B subclasses concentrations are included in total LDL cholesterol.

**Table 9:** Postprandial changes of LDL subclasses in men

**Lipoprotein**	**Cohort**	**Fasting**	**Postprandial**	**P (time)**	**P (time × waist)**
LDL (mg/dl)	Control	105.7 ± 32.05	94.33 ± 20.23	< 0.05	0.486
	Abdominal obesity	127.3 ± 27.54	117.8 ± 26.94	< 0.01	
LDL 1 (mg/dl)	Control	37.67 ± 11.31	35.56 ± 11.47	< 0.05	0.187
	Abdominal obesity	44.44 ± 8.002	40.10 ± 7.762	< 0.01	
LDL 2 (mg/dl)	Control	15.50 ± 15.31	14.5 ± 14.04	0.132	0.253
	Abdominal obesity	24.89 ± 11.95	26.03 ± 12.95	0.539	
LDL 3–7 (mg/dl)	Control	4.667 ± 12.16	2.778 ± 7.596	0.089	0.875
	Abdominal obesity	2.444 ± 2.833	3.444 ± 4.586	0.188	

**Table 10:** Postprandial changes of LDL subclasses in women

**Lipoprotein**	**Cohort**	**Fasting**	**Postprandial**	**P (time)**	**P (time x waist)**
LDL (mg/dl)	Control	104.00 ± 32.90	97.78 ± 32.71	< 0.05	0.377
	Abdominal obesity	106.70 ± 27.12	99.89 ± 26.56	< 0.05	
LDL 1 (mg/dl)	Control	37.22 ± 11.03	37.00 ± 10.9	1.000	0.285
	Abdominal obesity	42.33 ± 11.86	41.33 ± 12.18	0.440	
LDL 2 (mg/dl)	Control	8.89 ± 6.194	8.33 ± 4.500	0.888	0.324
	Abdominal obesity	14.67 ± 16.33	13.00 ± 12.77	0.438	
LDL 3–7 (mg/dl)	Control	0.78 ± 1.394	0.67 ± 2.000	1.000	0.682
	Abdominal obesity	0.00 ± 0.000	0.22 ± 0.667	0.879	

### HDL and their subclasses

**Figure 10:**
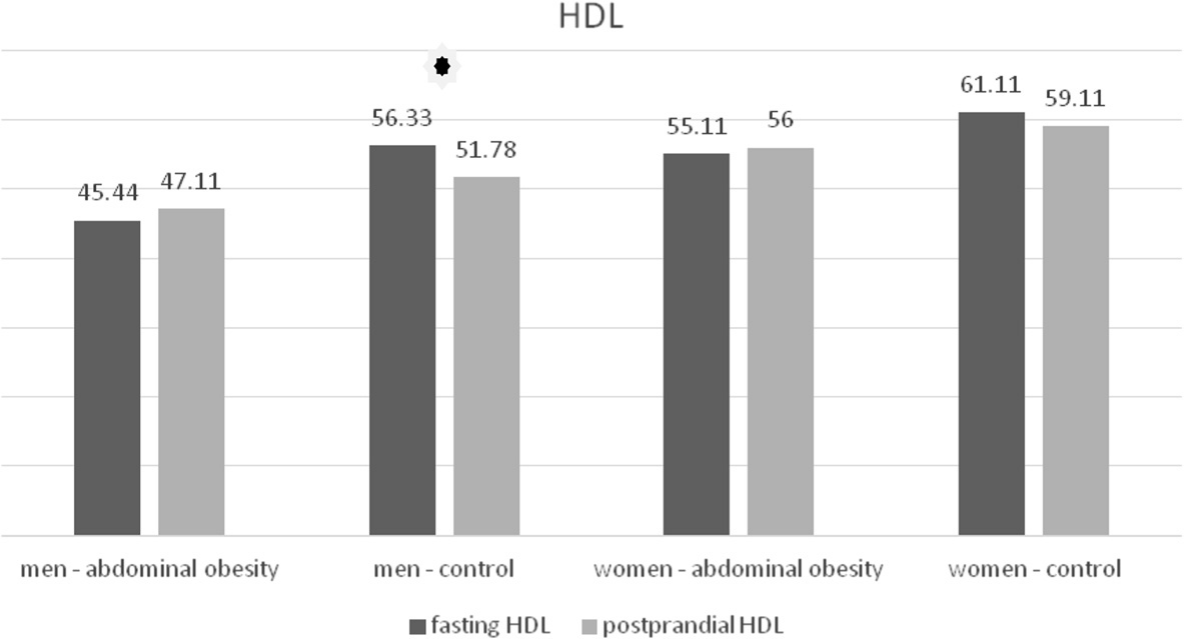
Fasting and postprandial total HDL concentrations.

**Figure 11:**
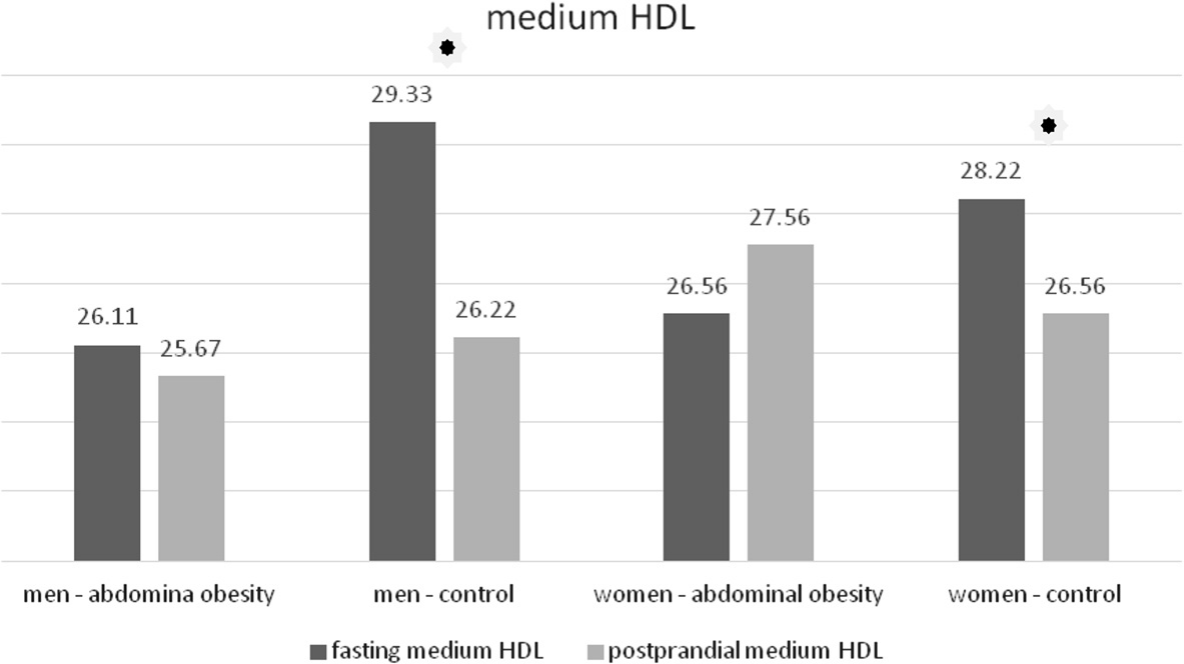
Fasting and postprandial medium HDL concentrations.

**Figure 12:**
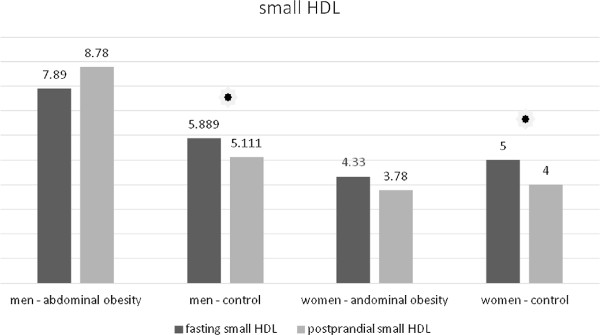
Fasting and postprandial small HDL concentrations.

**Figure 13:**
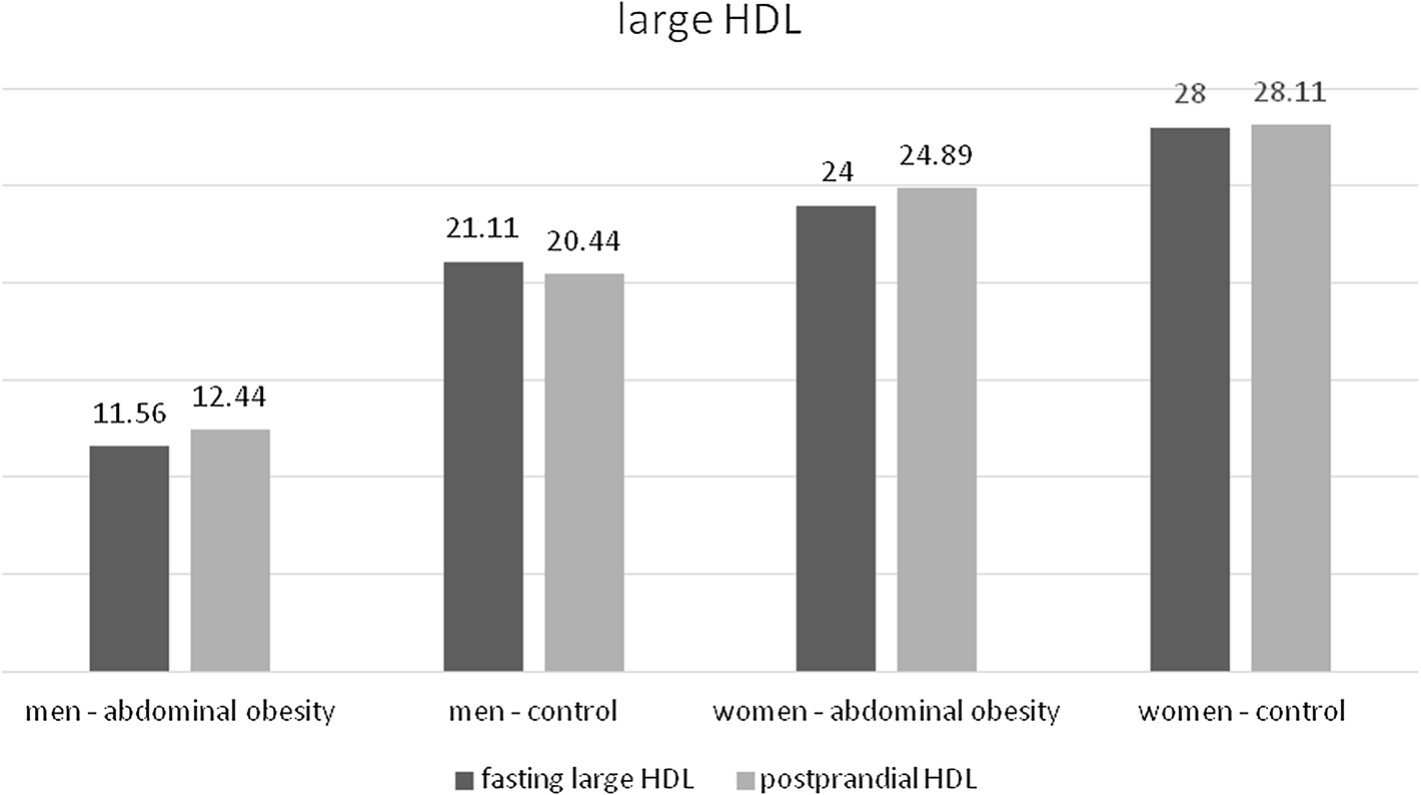
Fasting and postprandial large HDL concentrations.

In men without abdominal obesity there was a postprandial decrease of the total HDL concentration (Table 11, Figure 10). In women without abdominal obesity the decrease did not reach statistical significance, but there was a notable trend to decrease (p = 0.076). No statistically significant change occurred in the group of men and women with abdominal obesity (Table 12, Figure 10). In the analysis of HDL subclasses, we obtained similar results. In the subclasses of medium and small HDL particles their concentrations decreased in the group of the volunteers with normal waist circumference in both men and women (Tables 11 and 12, Figures 11 and 12). In the subclass of large HDL particles we did not note a statistically significant change in the concentration in any of the groups of volunteers (Tables 11 and 12, Figure 13).

**Table 11:** Postprandial changes of HDL subclasses in men

**Lipoprotein**	**Cohort**	**Fasting**	**Postprandial**	**P (time)**	**P (time × waist)**
HDL (mg/dl)	Control	56.33 ± 10.93	51.78 ± 7.902	< 0.05	< 0.05
	Abdominal obesity	45.44 ± 8.368	47.11 ± 7.737	0.858	
Large HDL (mg/dl)	Control	21.11 ± 7.976	20.44 ± 6.146	0.469	0.185
	Abdominal obesity	11.56 ± 5.981	12.44 ± 4.586	0.529	
Medium HDL (mg/dl)	Control	29.33 ± 3.279	26.22 ± 1.922	< 0.05	< 0.05
	Abdominal obesity	26.11 ± 3.444	25.67 ± 3.536	0.473	
Small HDL (mg/dl)	Control	5.89 ± 1.537	5.11 ± 1.364	< 0.05	< 0.05
	Abdominal obesity	7.89 ± 2.713	8.78 ± 5.472	1.000	

**Table 12:** Postprandial changes of LDL subclasses in women

**Lipoprotein**	**Cohort**	**Fasting**	**Postprandial**	**P (time)**	**P (time × waist)**
HDL (mg/dl)	control	61.11 ± 10.64	59.11 ± 10.48	0.076	< 0.05
	abdominal obesity	55.11 ± 9.545	56.00 ± 7.599	0.234	
Large HDL (mg/dl)	control	28.00 ± 10.75	28.11 ± 9.144	0.796	0.293
	abdominal obesity	24.00 ± 7.000	24.89 ± 6.451	0.202	
Medium HDL (mg/dl)	control	28.22 ± 3.632	26.56 ± 3.909	< 0.05	< 0.05
	abdominal obesity	26.56 ± 3.575	27.56 ± 3.575	0.618	
Small HDL (mg/dl)	control	5.00 ± 2.062	4.00 ± 2.121	< 0.05	< 0.05
	abdominal obesity	4.33 ± 1.394	3.78 ± 1.394	0.168	

## Discussion

We studied the acute (one-time) impact of high fat meal on the concentrations of serum lipoprotein classes and subclasses in healthy men and women as well as men and women with abdominal obesity. We observed significant postprandial changes in the lipoprotein profile across all lipoprotein particle classes. However, the individual volunteer groups differed from one another by the presence and extent of changes in certain lipoprotein classes and subclasses. Therefore we concluded that postprandial lipoprotein metabolism is strongly influenced by amount of visceral fat and gender.

### Triacylglycerols

The most apparent postprandial changes were observed in serum triacylglycerol concentrations. TAG increased postprandially in all subjects however the highest increase occurred in obese men. They had a more significant postprandial TAG rise than the other cohorts and concentration in abdominal obese men more than doubled. The cause of this phenomenon is apparently the significantly higher fasting TAG concentration in this cohort than in all other cohorts. It is known that the postprandial increase of TAG is strongly influenced by fasting triacylglycerolemia [6,7,16-18]. Postprandial TAG concentration in men with abdominal obesity was significantly higher than in non-obese men, but there was no statistically significant difference in postprandial TAG concentration between women with and without abdominal obesity. Although the mean fasting TAG concentration in abdominal obese women was also slightly higher than the mean fasting TAG concentration in women without abdominal obesity, this difference was not as apparent as in men (Tables 1 and 2). Huge postprandial increase in TAG concentration in obese men can be explained by reduced postprandial lipolytic activity, which has been described in individuals with abdominal obesity [14]. In our study we noted differences between men and women as regards the postprandial response given by the rise of TAG. While men’s waist circumference was a significant determinant which decides about the degree of TAG rise, we cannot conclude the same for the women. This result is apparently related to the significantly higher average fasting concentration of TAG in men with abdominal obesity compared with women with abdominal obesity.

### VLDL

In the case of VLDL particles, we observed similar results. Observed postprandial rise of VLDL corresponds with the other studies [6,13]. The rise of VLDL is apparently caused by their increased synthesis from exogenous triacylglycerols in the liver, which is stimulated by the postprandial insulinemia [19]. However, in the group of women with a normal waist circumference we did not note a statistically significant increase. Wojczynsk et al. observed a small but statistically significant postprandial increase in VLDL in the group of normotriacylglycerolemic women. The average waist circumference in this group was above the IDF normal level therefore we conclude that this group included also a significant proportion of women with abdominal obesity [6]. This fact may be the cause for different results in our study, whereas the composition of the groups compared were different. Observed higher increase in men with abdominal obesity is consistent with the finding of significantly higher mean fasting VLDL in this cohort. There was no significant difference in the fasting VLDL concentrations between the abdominal obese and non-obese women; however, women with abdominal obesity had higher postprandial TAG concentration.

### IDL and their subclasses

In the case of large IDL particles we observed a quite different trend than in the case of TAG. The concentration of large IDL particles increased after the meal in all groups, except in the group of men with abdominal obesity, although there was the highest increase in the concentration of VLDL in this group. We do not have a clear explanation for this phenomenon. One possible explanation may be the prolonged plasma half-life of VLDL in obese subjects noted in some previous studies [20]. Since large IDL particles are the product of VLDL catabolism, the prolonged VLDL half-life may indicate reduced catabolism of VLDL particles and hence a decreased in the production of IDL. In the subclass of medium IDL we noticed a little but statistically significant decrease after the meal. Similarly, a slight decrease of small IDL after the meal was observed in all groups except the group of women with abdominal obesity in which the decrease was at the limit of statistical significance. A similar work analyzing the postprandial changes in the IDL subclasses have not been carried out yet. The studies published to date recorded a postprandial increase in the total concentration of IDL with a peak at 2 hours after meal [21].

### LDL and their subclasses

In all groups we observed a small but statistically significant decline in the total LDL cholesterol concentration. Similar results have also been obtained by other authors [6,16]. It is the reduction of the MID A and MID B lipoprotein fractions, which is mainly responsible for the decrease in the LDL concentration in the study population. By the Quantimetrics Lipoprint LDL system the MID A and MID B subclasses concentrations are included in total LDL cholesterol. These particles have got lower electrophoretic mobility than the subclass of large LDL particles (LDL 1) and consist of small and medium IDL particles. In the analysis of LDL subclasses, we found a statistically significant decrease in the concentration of large LDL particles in men, but no change occurred in women. We also have not found any changes in postprandial concentrations of medium and small dense LDL particles. In the case of small dense LDL particles, these results are not relevant, as only 7 men and 3 women out of the 48 volunteers had detectable concentrations of small LDL particles. Greatest number of participants with detectable small dense LDL particles was in the cohort of men without abdominal obesity. In this group, the trend was toward a decrease in the postprandial concentration of small dense LDL, which, however, did not reach statistical significance. The reason for the low number of volunteers with detectable concentrations of small dense LDL is their selection. We selected young individuals with very low overall cardiovascular risk. Volunteers with a known history of cardiovascular disease, diabetes and dyslipidemia were excluded. To obtain relevant results, it would be necessary to implement work to include a greater number of volunteers and to focus on individuals with a high fasting concentration of LDL. Wojczynsk et al. reported a statistically significant postprandial decrease in the quantity of small dense LDL in both men and women. Their study included a large number of subjects with hypertriacylglycerolemia, who have got on average a higher concentration of small dense LDL [6]. On the contrary, Koba et al. who examined the postprandial changes of size of LDL particles in subjects who undergone myocardial infarction observed a decrease in their mean size peaking sixth hour after the meal [10].

### HDL and their subclasses

We observed differences between men and women in the postprandial response also in the case of HDL cholesterol. In study population, there was a postprandial decrease in the total concentration of HDL in the group of non-obese men. In non-obese women, there was a trend to decrease, which did not reach the statistical significance. Wojczynsk et al. recorded a slight decrease in postprandial HDL cholesterol in men and in women with normal concentration of TAG [6]. We do not know why HDL drops postprandially only in normotriacylglycerolemic and non-obese subjects, but not in subject with abdominal obesity. Decrease of total HDL cholesterol in non-obese subject is caused by decrease of subclasses of medium and small HDL particles. Wojczynsk et al. reported a postprandial decrease in the concentration of small HDL in all groups. On the contrary, however, their study recorded a rise of in the subclass of medium HDL particles [6]. Our study differed in the method of measurement of lipoprotein particle subclasses (electrophoresis in polyacrylamide gel versus nuclear magnetic resonance imaging), in the selection of subjects within groups as well as in the time of blood sampling after meal. All these factors could contribute to the incompatibility of our results. Nevertheless, we lack a clear explanation for this phenomenon.

### Limitations

Variations in activity of several enzymes involved in the lipoprotein metabolism occur during the postprandial period. These changes are probably reflected in the postprandial changes of lipoprotein profile. Postprandially, LDL and HDL are enriched with TAG by activity of CETP (cholesterylester transfer protein), which mediates the exchange of TAG for cholesterols between these particles and VLDL and chylomicron remnants [22]. Subsequently, such particles are a substrate for HL (hepatic lipase) [23]. Study by De Bruin et al. observed the postprandial changes in HDL concentration in healthy men, reported increased hepatic lipase activity, while the decrease in HDL correlated with the HL activity [11]. These mechanisms may be responsible for the postprandial decrease in HDL and LDL concentrations. The CETP-mediated interaction between VLDL and HDL and LDL, however, also leads to a change in the LDL and HDL composition in favor of small particles formation [24]. Parra et al. described negative correlation of postprandial VLDL concentration and CETP activity in healthy women [25]. VLDL increased more in the men and women with abdominal obesity so CETP activity in these subjects might be lower. We did not study activities of CETP or HL. The study of association of activity of these enzymes and lipoprotein concentration changes could provide us with better understanding of the postprandial lipoprotein metabolism.

### Complex meal versus lipid rich formula

In our study we decided to use a complex meal for the lipid load, formulated to imitate regular diet in the Western culture. We prefer this procedure before using any formulas high in fat and low in carbohydrates based on cream and oils used by some other authors. The reason was the effort to approach as close as possible the metabolic state, which is normally present after the regular meal. Some of the prior studies actually show that the individual nutrient composition of diet can significantly affect the postprandial lipoprotein metabolism [26].

### Effect of postprandial lipidemia on cardiovascular risk

High postprandial triacylglycerolemia is strong predictor of myocardial infarction and ischemic heart disease [5]. Therefore elevated postprandial TAG seen in men with abdominal obesity may participate in pathogenesis of premature cardiovascular event. Lower lipolytic activity described in subjects with abdominal obesity may be responsible for higher postprandial TAG and VLDL concentrations and no rise of large IDL particles observed in our study. VLDL interacts with LDL and HDL particles by activity of CETP and participates in small dense LDL formation and HDL degradation [8,23]. Whereas the VLDL concentrations are even higher in the postprandial state, we assume that these interactions are more extensive in this state as in fasting period. Therefore postprandial rise of VLDL may contribute in formation of dyslipidemia with accumulation of small dense LDL and low HDL in subject with abdominal obesity and metabolic syndrome. However, in our study population, we did not record postprandial increase of small dense LDL or decrease of HDL particles in subjects with abdominal obesity. As we mentioned before, this my however been caused by selection of low risk young volunteers with very low or none small dense LDL concentrations. So these interactions will require further study including volunteers with higher basal small dense LDL concentrations.

## Conclusions

We observed significant postprandial changes of the lipoprotein profile. These changes differ by the nature and extent depending on the presence of abdominal obesity. Therefore, we assume that postprandial lipoprotein metabolism is strongly influenced by the excessive accumulation of visceral fat. The men with abdominal obesity had significantly higher postprandial triacylglycerol concentration than non-obese men, although mean and median fasting TAG concentration in this cohort was within normal range. High postprandial TAG in abdominally obese men may participate in pathomechanism leading from visceral fat accumulation to accelerated atherosclerosis and premature cardiovascular event. The postprandial TAG elevation is accompanied by rise of VLDL concentration. Extent of postprandial VLDL increase did not differ significantly between men with and without abdominal obesity, however postprandial VLDL concentration was significantly higher in abdominally obese men. VLDL increased also in women with abdominal obesity, but there was any notable increase in non-obese women. High postprandial VLDL concentration may participate in the formation of small dense LDL particles and may increase the rate of HDL degradation. However, we did not record postprandial increase of small dense LDL or decrease of HDL particles in subjects with abdominal obesity. Large IDL particles which represent direct VLDL remnants rise only in volunteers with normal waist circumference. Lack of increase of VLDL remnants and high postprandial VLDL may indicate lower lipolytic activity in subject with abdominal obesity. We observed postprandial decrease of medium and small IDL particles. This has not yet been recognized before. Pathophysiological significance and biochemical causes of this phenomenon are not known and will require further investigation. We believe that our findings emphasize the importance of abdominal obesity and its relations to postprandial lipoprotein metabolism in the atherosclerosis pathogenesis pointing at the extensive rise of VLDL and TAG and possible lower lipolytic activity.

## Methods

### Participants

We recruited healthy volunteers with normal waist circumference and abdominal obesity aged from 21 to 40 years who signed informed consent.

### Exclusion criteria

The exclusion criteria comprised of smoking, alcohol or drug abuse, known history of diabetes mellitus, malignancy, chronic kidney disease and cardiovascular disease, pregnancy, menstruation, use of hypolipidemics, corticosteroids, and hormonal contraceptives, as found out using a questionnaire. Volunteers with fasting serum glucose concentration above 6 mmol/l, TSH concentration outside the reference range and glomerular filtration calculated by MDRD equation of less than 1.0 ml/s were excluded.

### Study design

On the day when the study was conducted, the volunteers had to be fasting, having consumed the last meal on the previous day at 18:00. Since then they could drink water only. They were not allowed to take any food or sweetened drinks. The following day, the volunteers were instructed and signed the informed consent and filled a questionnaire. Their height, weight and waist circumference was measured according to the IDF recommendations for europoid population. At 10:00 AM, the volunteers ate a standard meal (bun, cooked ground beef, emmenthaler cheese, tomato, onion, ketchup, mustard). The test meal contained 850 kcal, 44 g proteins, 52 g fats, of which were 20 g saturated fats, 11 g carbohydrates, and 4 g fiber. The tested persons had to eat their test meal in 20 minutes. After the meal, the blood has been sampled immediately by venepuncture using Vacutainer closed system for serum sampling (with no additives). Blood sampling was performed by trained nurse. Than the volunteers were instructed to avoid consuming any more food or sweetened drinks or perform any increased physical activity prior to second blood sampling. 4 hours after the meal second blood samples have been obtained to determine postprandial lipoprotein concentrations.

### Sample processing

After the blood sampling, the samples remained for 30 minutes at room temperature and subsequently supernatant was separated by centrifugation for 10 minutes at 3,000 rpm. Some part of the supernatant was used for the determination of serum concentration of glucose, creatinine, TSH, cholesterol and TAG. The second part of the supernatant was frozen at the temperature of −80°C for analysis of lipoprotein classes and subclasses. Samples were stored less than 30 days.

### Biochemical methods

The concentrations of VLDL, IDL, LDL and HDL classes and subclasses were determined by the linear electrophoresis in polyacrylamide gel (Quantimetrix Lipoprint LDL System and Quantimetrix Lipoprint HDL System, RendoBeach, USA). Both methods use electrophoresis in tubes filled with 3% polyacrylamide gel. 25 μl of serum was mixed with a 200 μl of a solution of the liquid gel and sudan black, and added to the gel tube. Polymerization of the gel followed for 30 minutes at room temperature. Subsequently, electrophoresis was performed for 1 hour (3 mA/1 tube). After the electrophoresis took place, it was followed by densitometric reading and conversion to concentrations of lipoprotein classes and subclasses using the Lipoware software (Quantimetrix, RendoBeach USA). The Quantimetrix Lipoprint LDL System divides lipoproteins based on electrophoretic mobility and determines the concentration of VLDL, large, medium and small IDL particles, large LDL particles (LDL 1), medium LDL particles (LDL 2), and small dense LDL particles (LDL-3-7). The Quantimetrix Lipoprint HDL System method determines 10 HDL subclasses divided into large, medium and small HDL.

### Statistical methods

The descriptive data were provided as a ± standard deviation of average. The differences between the values of the parameters before and 4 hours after the meal were compared using the paired T-test for parameters with a normal distribution, or the Wilcoxon paired test to compare parameters without normal distribution. The delta values of lipoprotein concentrations before and after a meal were compared between different groups of volunteers using the unpaired T-test or Mann–Whitney unpaired nonparametric test according to distribution of each parameter. The D’Agostino-Pearson test and Kolmogorov-Smirnov test were used to verify the normal distribution of parameters in the cohorts. The normally distributed parameters were considered to be those which have passed the verification in both tests. A difference with the P-value of less than 0.05 was considered as statistically significant.

### Ethics committee

The authors declare that the study was approved by the Ethics Committee of Faculty of Medicine, Comenius University in Bratislava and was conducted in accordance with Declaration of Helsinki.

## Abbreviations

CETP: Cholesterylester transfer protein; HDL: High-density lipoprotein; HL: Hepatic lipase; the IDF: International diabetes federation; IDL: Intermediate-density lipoprotein; LDL: Low density lipoprotein; MDRD: Modification of diet in renal disease; TAG: Triacylglycerol; TSH: Thyroid stimulating hormone; VLDL: Very-low density lipoprotein.

## Competing interests

The authors declare that they have no competing interests.

## Authors’ contributions

PS was the main study designer, drafted the manuscript and participated in the statistical analysis. MC was the main manuscript reviser and participated in the statistical analysis. LG and PK were critical participants in study design and provided input within their respective areas of expertise during analysis and manuscript drafting and revision. MB and DB participated in the statistical analysis and manuscript revision. SO and AD oversaw the whole study realization, participated in study design and findings interpretation. All authors read and approved the final manuscript.
